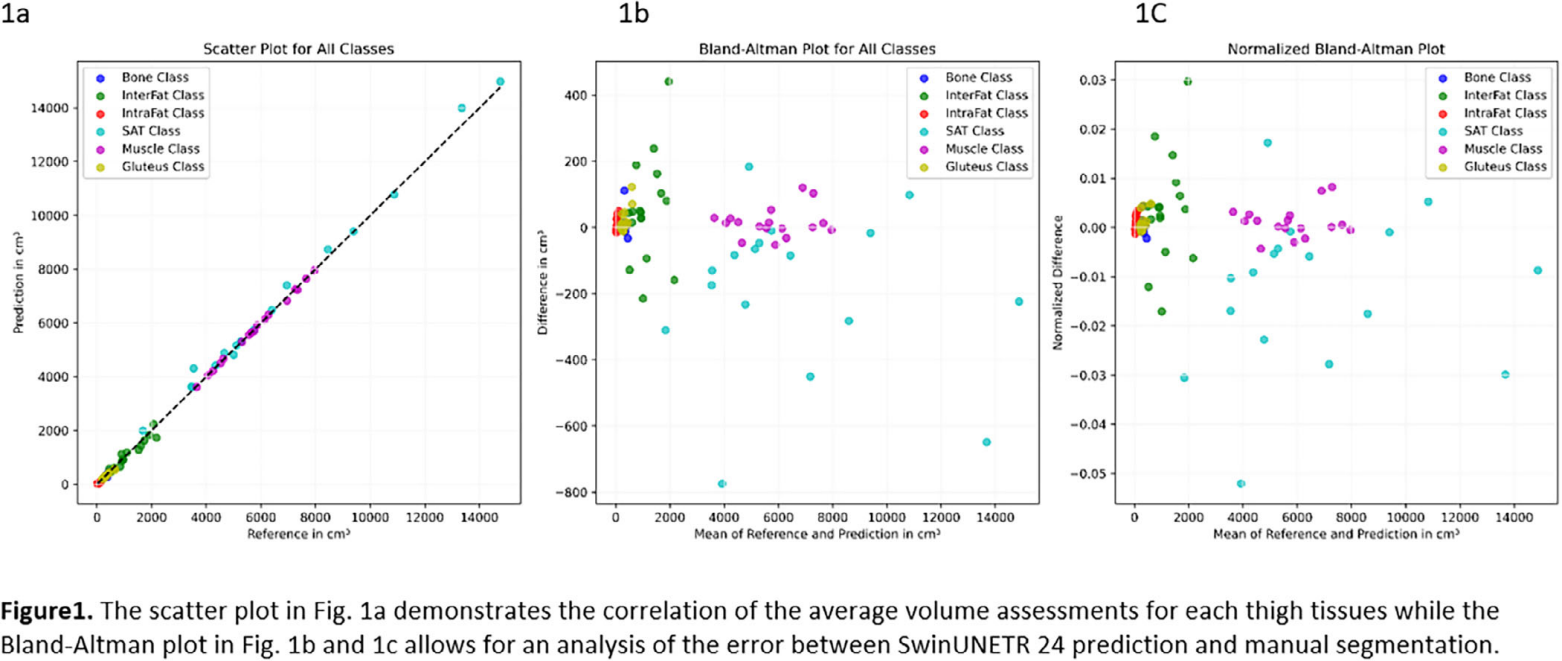# Automated Thigh Tissue Segmentation by Vision Transformers: Application for Early Alzheimer's Diagnosis

**DOI:** 10.1002/alz.092665

**Published:** 2025-01-09

**Authors:** Sara Hosseinzadeh Kasani, Mahsa Dolatshahi, Mahshid Naghashzadeh, Paul K. Commean, Farzaneh Rahmani, Jingxia Liu, LaKisha Lloyd, Caitlyn Nguyen, Nancy Hantler, Abby McBee‐Kemper, Maria Ly, Gary Z Yu, Joseph E. Ippolito, Bettina Mittendorfer, Claude Sirlin, John C. Morris, Tammie L.S. Benzinger, Cyrus A. Raji

**Affiliations:** ^1^ Mallinckrodt Institute of Radiology, Washington University in St. Louis, St. Louis, MO USA; ^2^ Washington University in St. Louis School of Medicine, St. Louis, MO USA; ^3^ Washington University in St. Louis, St. Louis, MO USA; ^4^ Missouri University School of Medicine, Columbia, MO USA; ^5^ University of California, San Diego, La Jolla, CA USA; ^6^ Knight Alzheimer Disease Research Center, St. Louis, MO USA; ^7^ Washington University School of Medicine in St. Louis, St. Louis, MO USA

## Abstract

**Background:**

Within the research field of neurodegenerative disorders, unbiased analysis of body fat composition, particularly muscle mass, is gaining attention as a potential biological marker for refining Alzheimer’s disease risk. The objective of this study was to employ a deep learning model for fully automated and accurate segmentation of thigh tissues, potentially contributing to early Alzheimer's diagnostics.

**Method:**

In an IRB‐approved study, 49 participants underwent thigh Dixon MRI scans with a TR=9.99s, TE=2.46s, flip angle=10°, and slice thickness= 5mm. The Dixon Fat/Water images were semi‐automatically segmented by an expert operator in all available slices to obtain the bone, intermuscular fat (InterFat), intramuscular fat (IntraFat), Subcutaneous Adipose Tissue (SAT), Muscle, and Gluteus. We trained and compared the performance of baseline and state‐of‐the‐art deep neural networks, namely, UNet, VNet, and two vision transformers (ViTs): UNETR and SwinUNETR. The performance of the trained models was tested on all data sets using a 3‐fold cross‐validation scheme.

**Result:**

We found SwinUNETR outperformed the others with a mean dice similarity coefficient 96.20 (± 0.51), 80.91 (± 0.55), 50.56 (± 1.43), 95.26 (± 0.80), 98.70), 86.72 (± 1.12) in Bone, InterFat, IntraFat, SAT, Muscle, and Gluteus, respectively. Bland–Altman analysis and scatter plot (Figure 1) indicated that the differences between manual annotations and predictions by the SwinUNETR model were relatively minor for Bone volume, Intramuscular Fat volume, Muscle volume, and Gluteus volume classes. The overall mean difference is ‐88.8cm^3^ with a 95% confidence interval (CI) of [‐159.53, ‐18.13]. Biases [95% CI] for each tissue class were 5.44cm^3^ [−8.61, 19.50] for Bone volume, 51.74cm^3^ [−22.72, 126.20] for InterFat volume, 11.15cm^3^ [2.76, 19.53] for IntraFat volume, ‐191.09cm^3^ [−309.54, ‐72.64] for SAT volume, 15.16cm^3^ [−6.37, 36.70] for Muscle volume, and 18.76cm^3^ [2.20, 35.32] for Gluteus volume.

**Conclusion:**

This study highlighted the use of ViTs for the automated segmentation of thigh tissues in MR which may allow for the detection of subtle changes in muscle mass and fat composition, that are of increasing interests in their associations with the neurodegenerative processes in Alzheimer's disease.